# Supplementary material for: Development of antigen multimers for detection and evaluation of CAR T cells
Source: Cancer Immunol Immunother. 2025 Oct 23;74(11):344. doi: 10.1007/s00262-025-04134-9 (PMC12550087; doi:10.1007/s00262-025-04134-9)
Supplement: Supplementary file 1 — (DOCX 793 KB) [file 262_2025_4134_MOESM1_ESM.docx]

Supplementary figures


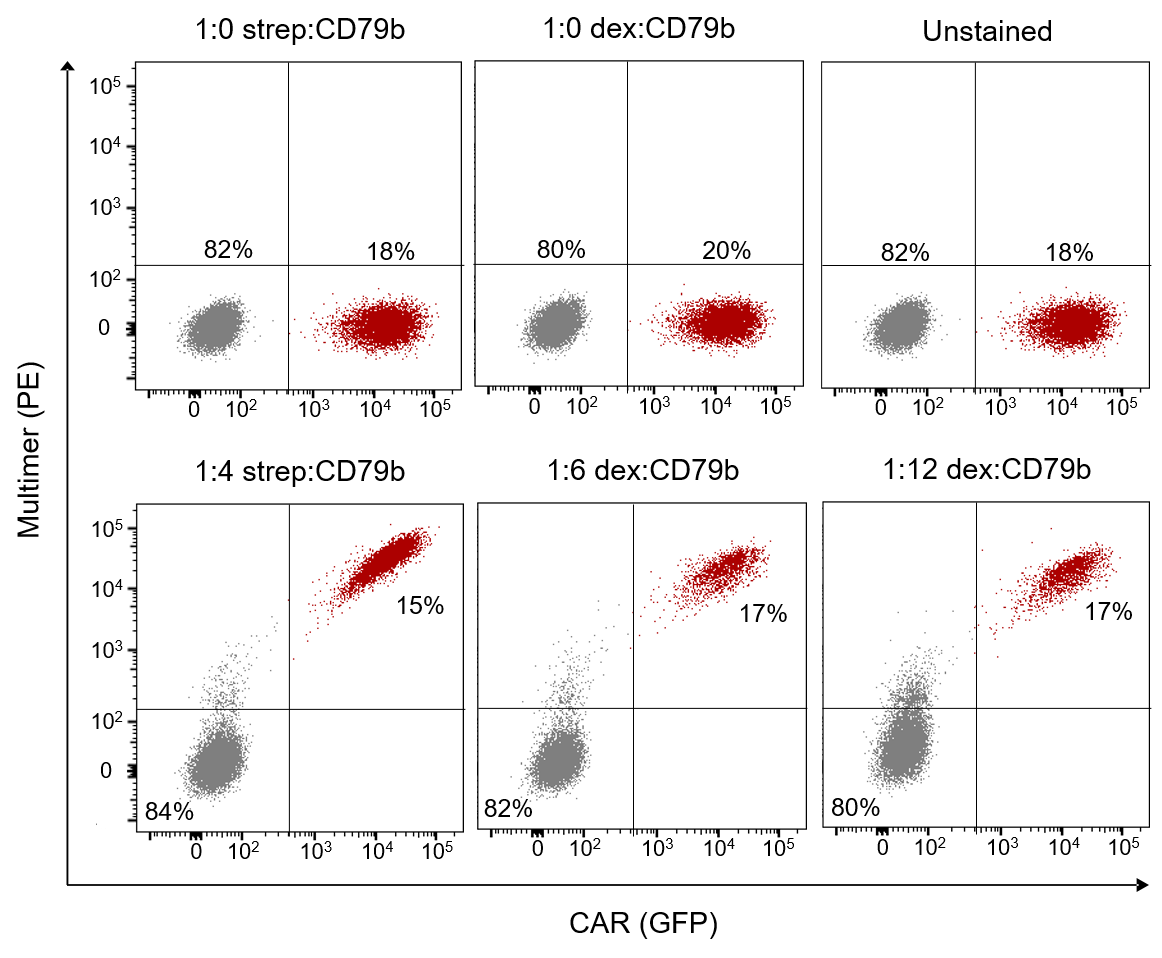


Supplementary Figure 1. Antigen-multimer staining of anti-CD79b CAR. Anti-CD79b CAR-expressing SupT1 cells stained with PE-labelled CD79b-tetramer and two different CD79b-multimers. Unstained cells and PE-labelled streptavidin or dextran backbones were used as controls.


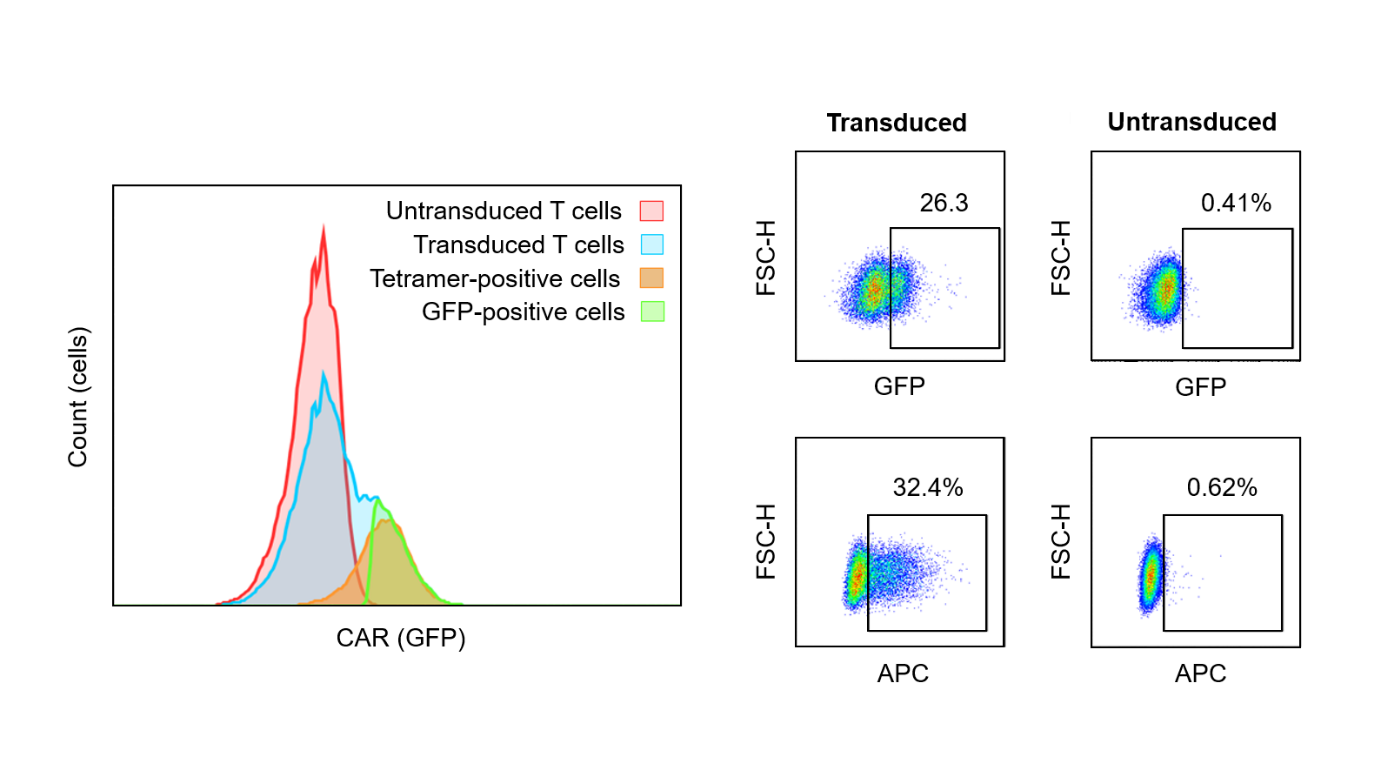


Supplementary Figure 2. Antigen-tetramers detect low levels of CAR expression in T cells. Comparison between GFP- and tetramer-based detection of anti-CD19 CAR-expressing T cells transduced with a low MOI of lentivirus. Gating was done manually using the FlowJo software.


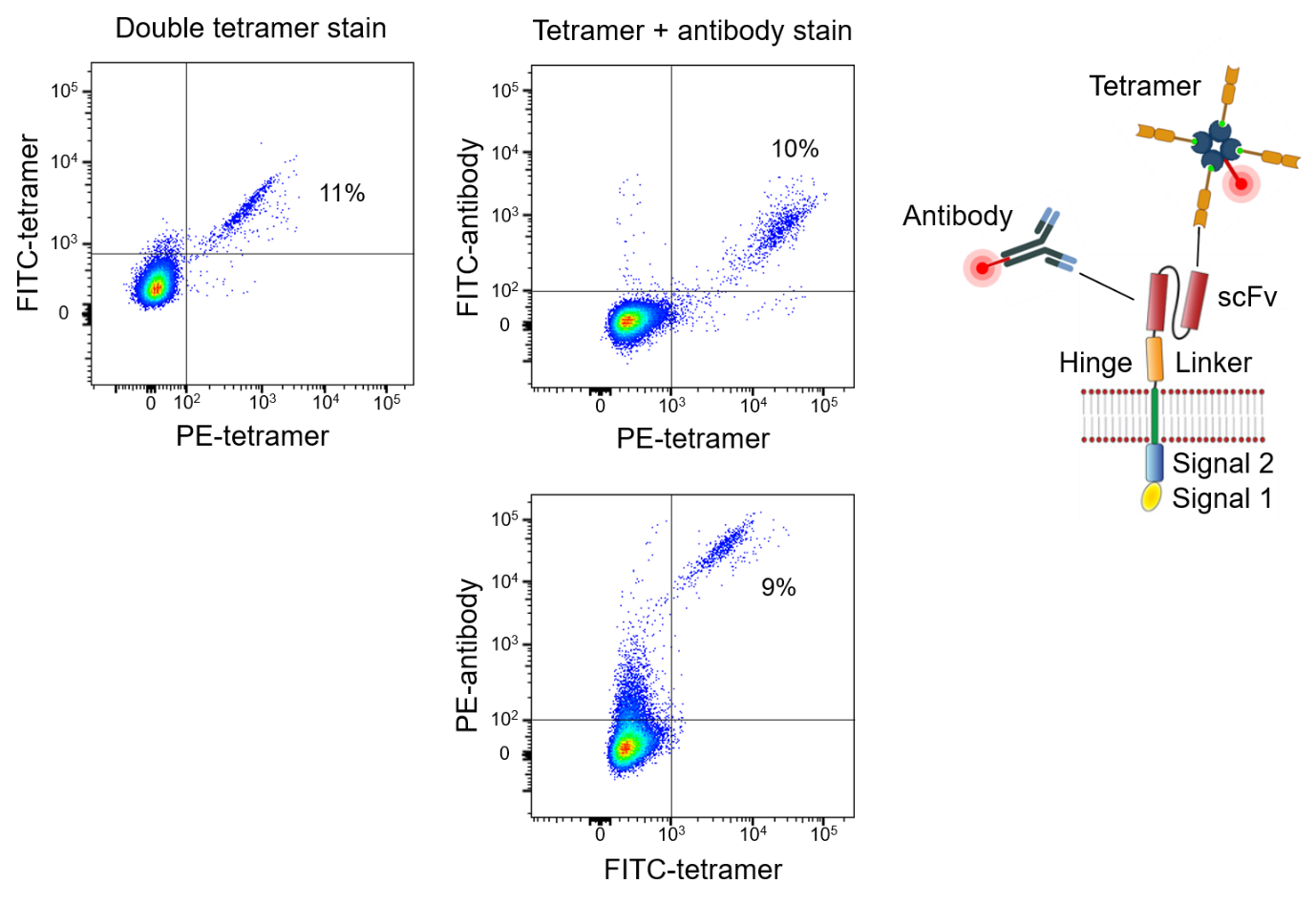


Supplementary Figure 3. Antigen-tetramers can co-stain CARs with anti-IgG antibodies. Staining of anti-Her2 CAR-expressing Jurkat cells demonstrating simultaneous binding of Her2-tetramers and anti-IgG antibodies.


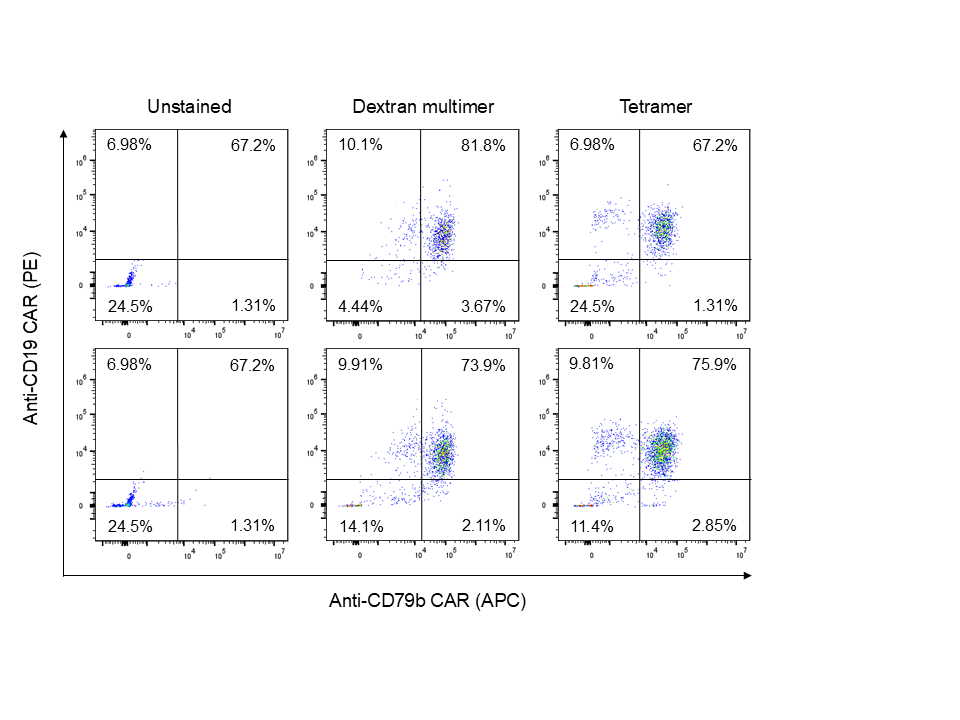


**Supplementary Figure 4. Antigen-multimers stain Jurkat cells co-expressing two different CARs.** Jurkat cells co-expressing anti-CD79b and anti-CD19 CARs stained with PE-labelled CD19-multimers and APC-labelled CD79b-multimers. Unstained cells were used as controls.


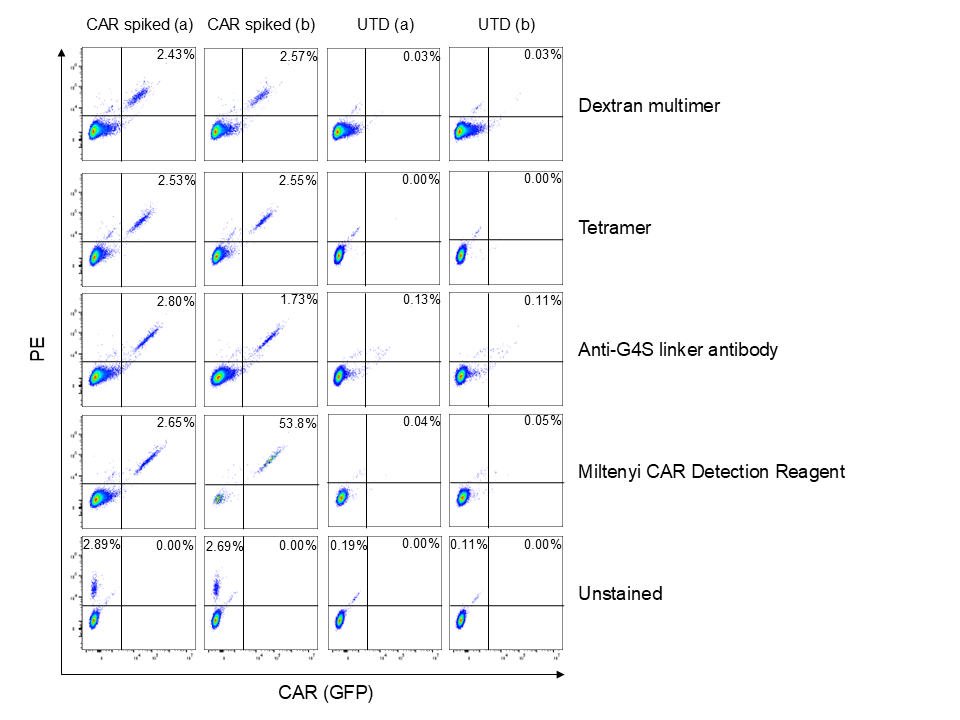


**Supplementary Figure 5. Antigen-multimers compared to commercially available reagents.**Comparison between PE-labelled staining reagents for detection of anti-CD19 CAR-expressing Jurkat cells. CAR-expressing cells were spiked into CAR-negative Jurkat cells and staining of untransduced (UTD) cells was used as negative control.
